# Comparison Between Menstrual Migraine and Menstrual-Unrelated Migraine in Women Attending Gynecology Clinics

**DOI:** 10.7759/cureus.10976

**Published:** 2020-10-16

**Authors:** Mohammad Abdullah, Sara Qaiser, Ayesha Malik, Manahil Chaudhry, Tehreem Fatima, Ayesha Malik, Noreena Iqbal

**Affiliations:** 1 Medicine, Combined Military Hospital Lahore Medical College & Institute of Dentistry, Lahore, PAK; 2 Medicine, Hameed Latif Hospital, Lahore, PAK; 3 Internal Medicine, Milton Keynes University Trust Hospital, Milton Keynes, GBR

**Keywords:** migraine, menstrual migraine, menstrual unrelated migraine

## Abstract

Background

Migraine is amongst the top 10 most disabling conditions, and the disease burden is highest in young and middle-aged women. Another variant of the migraine headache, menstrual migraine (MM) is experienced by this cohort of patients. Former studies have done comparisons between various demographic and clinical features of MM versus menstrual-unrelated migraine (MUM) in patients presenting to various clinics with the primary complaint of headaches. We aimed to compare symptoms of migraine in women attending gynecology outpatient clinics, regardless of their presenting complaint. This would help lessen the selection bias and produce more generalizable results.

Methods and materials

A cross-sectional study was conducted in the outpatient gynecology clinics at a tertiary care hospital over six months. The clinic attendees were screened for sufferers of a primary headache of the migraine type. The migraineurs were then stratified into groups A, MM patients, and group B, MUM patients, using the International Headache Society (HIS) criteria. They were then questioned for the presence of various symptoms associated with their migraine attacks for comparison.

Results

One-hundred eighty-one women (between 12 years to 55 years) were found to have primary headaches; amongst these, 126 patients met the inclusion criteria and consented to participate; from these, 62 (49.2%) patients had MM and 64 (50.8%) patients had MUM. The symptoms of nausea (p=0.00269), photophobia (p=0.000088), and phonophobia (p=0.0281) were statistically higher in MM patients while vomiting was not a significant feature. Both groups had a predominantly unilateral headache. The average days of the attack had a significant difference between the two groups (p=0.000019), where the duration was longer for MM patients.

Conclusions

It was observed that patients with MM tend to experience more features associated with migraine headaches, including a longer duration of attacks, and have a worse experience overall.

## Introduction

Migraine is amongst the top 10 most disabling conditions and the disease burden is highest in young and middle-aged women [1]. It not only leads to work and school absenteeism but also reduces productivity and adversely affects the quality of life (QOL) [2]. Menstrual migraine (MM) is defined as a migraine attack that occurs predominantly during an interval from two days before to three days after the onset of menstruation in at least two of three menstrual cycles. It is subdivided into a pure menstrual migraine (PMM) and menstrually related migraine (MRM) [3]. MRM is defined as attacks that occur in the peri-menstrual period but can occur at other times of the cycle as well while PMM is diagnosed when attacks are purely confined to the peri-menstrual period.

The pathogenesis for menstrual migraine is associated with estrogen withdrawal [3]. MM is usually more severe, longer-lasting, and more resistant to treatment due to the effects of ovarian hormones as compared to menstrual-unrelated migraine (MUM) [4].

The reported prevalence of menstrual migraines varies greatly, and the present literature is scanty. Furthermore, previous literature has assessed mainly patients with a primary complaint of headache, which may cause a selection bias. Moreover, patients with less severe symptoms and those not seeking medical advice are missed, hence this can decrease the external validity of the results. Our study aimed to compare the frequency of migraine-associated features between the two groups - those with MM and those with MUM in women attending gynecology clinics.

## Materials and methods

Following approval from the Ethics Review Committee, a cross-sectional study was carried out at a tertiary care hospital’s gynecology outdoor patient department (OPD) over six months, from January 2020 to June 2020. All clinic attendees, regardless of their presenting complaint, were screened for primary headaches via a non-probability, consecutive method. Informed consent was sought, and all women aged 18 years and above who consented to participate were included. All participants were then interviewed by a doctor to determine whether the headache was of the migraine type or not, i.e., whether it met the International Classification of Headache Disorders (IHCD-3) criteria for migraine [5] or if the patient had a prior diagnosis of migraine or was on migraine prophylaxis. Patients with migraine who met the inclusion criteria proceeded with filling a questionnaire based on the IHCD-3 criteria to assess for the presence of menstrual migraine. Patients with primary headaches who were non-migraineurs, those with amenorrhea, and those who failed to give consent were excluded.

Those who completed the questionnaire were then stratified into two groups. The first group (Group A) included those patients with menstrual migraine (MM), based on the IHCD-3 definition of menstrual migraine. The second group included all cases of migraines who experienced episodes at other times unrelated to the menstrual cycle and were labeled as cases of MUM.

Based on a thorough history, the following features of the headaches were noted:

1. Duration of the attack

2. Average days of the attack

3. The occurrence of associated symptoms (nausea, vomiting, photophobia, phonophobia)

4. Site of headache (unilateral or bilateral)

Demographic data, symptoms of migraine, some aspects of gynecological history (dysmenorrhea, age at menarche), and drug history were recorded and data analyzed using Statistical Package for the Social Sciences (SPSS) v25 (IBM Corp., Armonk, NY). Descriptive statistics were drawn for continuous data and inferential statistics determined for various features of the two groups. Demographic characteristics and other continuous data were presented as means and standard deviations. A chi-square test was used to compare nominal data, and the independent two-tailed t-test was used for the comparison of means of all normally distributed variables. For all statistical tests, the confidence interval was set at 95% and a p-value of ≤0.05 was considered significant.

## Results

From January to June 2020, 181 women of South Asian-Pakistani ethnicity were screened in the gynecology outpatient department (OPD) and were found to have primary headaches. One-hundred twenty-six (69.6%) patients met the inclusion criteria and were diagnosed with a primary headache of the migraine type. Group A comprised 62 (49.2%) patients with MM as per the IHS criteria while Group B comprised 64 (50.8%) patients who were diagnosed with MUM. The age range of all selected patients was between 12 and 55 years of age with a mean age of 31.99±8.05 years. There was a statistically significant difference (p=0.000647), such that patients with MUM had a lower age (29.63 years) as compared to MM patients with a mean of 34.44 years.

The age of menarche had no significant difference in the two groups with a mean age of 12.6 ± 1.21 years for MM patients and 12.5 ± 1.18 years for MUM patients. Coming to the features of the headache, the average days of the attack had a significant difference amongst the two groups (p=0.000019), with the duration being longer for MM patients. That is, the average days of attack for MM patients was 2.71 days as compared to 1.81 days for MUM patients.

Various symptoms of migraine were compared between the two groups (Tables 1-2). Nausea was significantly more prevalent in patients with MM, at 87.1%, as compared to MUM patients, at 64.1% (p=0.00269). Similarly, photophobia (p=0.000088) and phonophobia (p=0.0281) were also a more significant feature of MM. Lastly, both groups had a predominantly unilateral headache with 77.4% and 59.4% in MM and MUM patients, respectively. On the other hand, 40.6% of MUM patients had a bilateral headache as compared to 22.6% in MM patients. Nonetheless, this was a statistically insignificant association (p=0.604).

**Table 1:** Comparison of demographic features between MM and MUM MM: menstrual migraine; MUM: menstrual-unrelated migraine

	Menstrual Migraine		Menstrual-Unrelated Migraine		P-Value Two-tailed independent T-test
	Mean	Standard Deviation	Mean	Standard Deviation	
Age (years)	34.44	9.47	29.63	5.50	0.000647
Age at Menarche (years)	12.60	1.21	12.5	1.18	0.650
Average days of attack (days)	2.71	1.26	1.81	0.99	0.000019

**Table 2:** Comparison of symptoms of migraine between MM and MUM MM: menstrual migraine; MUM: menstrual-unrelated migraine

	Menstrual Migraine (MM) (n=62)	Menstrual-Unrelated Migraine (MUM) (n=64)	P-Value (<0.05) Chi-Square Test
Nausea	54 (87.1%)	41 (64.1%)	0.00269
Vomiting	39 (62.9%)	34 (53.1%)	0.266
Photophobia	42 (67.7%)	21 (32.8%)	0.0000880
Phonophobia	27 (43.5%)	16 (25%)	0.0281
Site	Unilateral – 48 (77.4%) Bilateral – 14 (22.6%)	Unilateral - 47 (59.4%) Bilateral - 17 (40.6%)	0.604

## Discussion

Our study was a comparison between the symptoms of migraine as well as certain demographic characteristics amongst patients with MM and MUM, screened and stratified in a gynecology outpatient clinic. This was different from studies done by Granella et al. [6] and MacGregor et al. [7] who chose their study sample from headache centers and miscellaneous primary care centers, respectively. The phenomenon of menstrual distress may lead to an exaggeration of symptoms in patients who have the chief complaint of such headaches, which was recognized as a limitation in a study by Pinkerman et al. [8]. Therefore, the aim of selecting samples randomly from gynecology clinics was to eliminate the selection bias that can occur with patients primarily presenting with headaches and produce generalizable results, regardless of the intensity of the headache.

Our study included women with a mean age of about 32 years who were all of the Asian ethnicity, while in another analogous study by MacGregor et al. [7], the mean age of women, predominantly Caucasian, of their sample was 37.6 years. Furthermore, in our study, the mean age of the two groups differed in a statistically significant way such that the cohort of MM patients had a younger age, at 30 years, while the mean age of patients with MUM was about 34 years (p=0.000647).

In addition to age, the symptoms of migraine, namely, nausea, vomiting, photophobia, and phonophobia were compared. The results revealed that while nausea (p=0.00269) was statistically higher in the MM group, the presence of vomiting while being more prevalent in MM patients was a statistically insignificant finding. Furthermore, photophobia (p=0.000088) and phonophobia (p=0.0281) were significantly more prevalent in MM patients. A systematic review by Vetvik et al. [9] elaborated that only two out of six studies who compared various migraine features between MM and MUM found a statistical difference of more nausea and vomiting in MM patients, similar to our study. However, this is contrary to what Granella [6] and Diamond [10] and their colleagues observed in their clinical studies, which had no difference in associated symptoms between the two groups. However, two prospective studies based on a diary-confirmed International Classification of Headache Disorders, Second Edition (ICHD-2) diagnosis of menstrual migraine did support our findings [7,11].

In terms of headache duration, our study showed that MM attacks lasted longer than MUM attacks (p=0.000019), as also concluded by Pinkerman et al. [8]. Longer attack days mean poorer quality of life, work absenteeism, and lower productivity. A comparison of the duration of the headache attacks was preferred over comparing the intensity of headache in our study, as a headache wasn't the main presenting complaint of patients.

Our study did, however, have some limitations. Due to the lack of local epidemiological data on disease burden, it’s difficult to estimate an adequate sample size to reflect the findings on the actual population, hence we sampled participants consecutively over the defined study period. Many women, particularly in our South Asian population, have an evasive attitude toward questions on menstruation and this influences their reporting of menstrual-related problems, including migraine. Several clinics and population diary studies rarely had a chance for three months follow-up, hence it is uncertain whether women who fulfill diagnostic criteria for MM in these studies occasionally experience attacks outside of their menstrual window. Hence, further longitudinal studies are needed to clarify this. Data from a migraine diary, collected over several months, would have been the ideal means of classifying women. Since this data was not available, we relied on self-reported data. In our study, we did not emphasize treatment, whether short-term prophylaxis or any other form of therapy, because these clinic attendees were not primarily being brought to the hospital for headaches.

## Conclusions

There is contradictory evidence as to whether various migraine features occur more frequently in MM patients or not. However, most of this data is derived from either headache clinics, which may result in selection bias, or from migraine diaries, which may result in recall bias or Hawthorne bias. To keep these biases at bay, we concluded that in terms of clinical features, patients with MM tend to experience more features of the migraine symptom complex and have a worse experience overall.
